# Insights into the regulation of wild soybean tolerance to salt-alkaline stress

**DOI:** 10.3389/fpls.2022.1002302

**Published:** 2022-10-19

**Authors:** Xiaoxi Cai, Bowei Jia, Mingzhe Sun, Xiaoli Sun

**Affiliations:** Crop Stress Molecular Biology Laboratory, College of Agriculture, Heilongjiang Bayi Agricultural University, Daqing, China

**Keywords:** wild soybean, salt stress, carbonate stress, ion balance, ROS scavenging, protein kinase, transcription factor

## Abstract

Soybean is an important grain and oil crop. In China, there is a great contradiction between soybean supply and demand. China has around 100 million ha of salt-alkaline soil, and at least 10 million could be potentially developed for cultivated land. Therefore, it is an effective way to improve soybean production by breeding salt-alkaline-tolerant soybean cultivars. Compared with wild soybean, cultivated soybean has lost a large number of important genes related to environmental adaptation during the long-term domestication and improvement process. Therefore, it is greatly important to identify the salt-alkaline tolerant genes in wild soybean, and investigate the molecular basis of wild soybean tolerance to salt-alkaline stress. In this review, we summarized the current research regarding the salt-alkaline stress response in wild soybean. The genes involved in the ion balance and ROS scavenging in wild soybean were summarized. Meanwhile, we also introduce key protein kinases and transcription factors that were reported to mediate the salt-alkaline stress response in wild soybean. The findings summarized here will facilitate the molecular breeding of salt-alkaline tolerant soybean cultivars.

## Introduction

Soybean is a world-widely grown crop, providing vegetable oils and proteins. Although soybean yield per acre has increased by approximately 40% during the past 20 years, current soybean production in China is still in severe shortage along with the population increase (USDA, 2021). On the other hand, roughly over 6% of the world’s soil resources are affected by saline and alkaline (data from GSASmap- https://www.fao.org/global-soil-partnership/gsasmap/en/). It is an effective way to expand the total soybean production by planting salt-alkaline tolerant soybean cultivars in the salt-alkaline soils. Therefore, it is an urgent demand to breed soybean cultivars with salt-alkaline tolerance.

Cultivated soybean (*Glycine max* L. Merr.) is domesticated from wild soybean (*Glycine soja* Siebold & Zucc.). Only approximately 50% of genes in *G. soja* are selected during long-term domestication and improvement, and many important genes related to environmental adaptation are lost (Kofsky et al., 2018). The narrow genetic variation in cultivated soybean seriously restricts the improvement of salt-alkaline stress tolerance. *G. soja* retains a higher level of genetic diversity and better adaptation to harsh environments (Kofsky et al., 2018). An effective way to breed stress-tolerant cultivars is to retrieve the stress tolerant genes from wild soybean and reintroduce them into cultivated soybean.

During the past decades, an increasing number of studies have demonstrated that plant cells were severely damaged by the primary ionic toxicity and secondary oxidative damage under salt-alkaline stress (Das and Roychoudhury, 2014). Therefore, researchers have made many efforts to identify genes involved in ion balancing and ROS scavenging (Zhao et al., 2021). Furthermore, current studies in the model plant Arabidopsis have strongly suggested that protein kinases and transcription factors are of paramount importance in governing the signaling transduction of plant adaptation to salt-alkaline stress (Chen et al., 2021a; Pan et al., 2022).

This review summarized the current research progress regarding the salt-alkaline stress response in *G. soja*. In this review, we summarized the functionally characterized genes in *G. soja* that regulated the capacity to the ion toxicity and ROS damage caused by salt-alkaline stress. We also introduced the protein kinases and transcription factors in *G. soja* that modulated the salt-alkaline tolerance. The findings summarized here will give soybean breeders an overall impression concerning the functional characterization of crucial salt-alkaline tolerant genes in *G. soja*, and will facilitate to illustrate the regulatory mechanism and signaling transduction pathways, as well as the molecular breeding of salt-alkaline tolerant soybean cultivars.

## Regulation of ion toxicity under salt-alkaline stress in wild soybean

**Figure 1:**
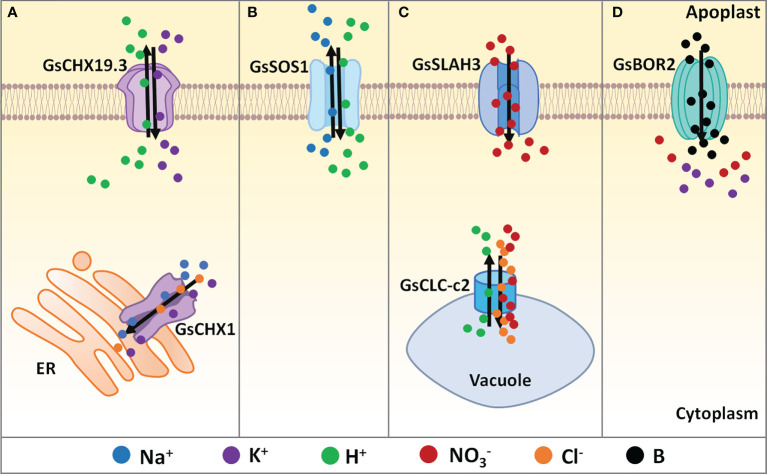
Regulation of ion balance under salt-alkaline stress in wild soybean. **(A)** Cation/H^+^ exchangers, **(B)** Na^+^/H^+^ antiporters, **(C)** Anion channels, **(D)** Boron transporters identified in *G. soja* involving in ion balancing under salt-alkaline stress. ER, endoplasmic reticulum. Solid dots with different colors represent different types of ions. Black arrows mean the direction of ion flow across the membrane.

Under salt stress, ionic toxicity caused by excess Na^+^ and Cl^-^ is the direct and primary damage to plants. Excess Na^+^ accumulation in plants further affects the uptake of other ions (such as K^+^) and causes osmotic stress, leading to water deficit inside plant cells (Assaha et al., 2017). Hence, balancing cytoplasmic ion status is very important for plants to survive under salt-alkaline stress. In this review, we summarized the genes (**Table 1**) and regulatory mechanism (**Figure 1**) of ion balance in *G. soja* response to salt-alkaline stress.

**Table 1:** Genes involved in ion balance under salt-alkaline stress in wild soybean.

Gene name	Gene ID	ID for *G. max* Homolog	Gene description	Function description
*GsCHX1/SALT3/Ncl*	Glyso.01G005509	Glyma.03G171600	Cation/H^+^ exchanger	It confers salt tolerance in soybean (Guan et al., 2014; Qi et al., 2014; Do et al., 2016; Liu et al., 2016; Lee et al., 2018; Qu et al., 2021).
*GsCHX19.3*	Glyso.17G039900	Glyma.17G043400	Cation/H^+^ exchanger	Its overexpression in Arabidopsis confers salt and bicarbonate alkaline tolerance (Jia et al., 2017).
*GmsSOS1*	Glyso.08G084400	Glyma.08G092000	Na^+^/H^+^ antiporter	Its overexpression in Arabidopsis confers salt tolerance (Nie et al., 2015).
*GsCLC-c2*	Glyso.16G165500	Glyma.16G208400	Chloride channel	Its overexpression in Arabidopsis and soybean composite plants confers chloride/salt tolerance (Wei et al., 2019; Liu et al., 2021b).
*GsSLAH3*	Glyso.10G192900	Glyma.10G229300	Slow-type anion channel homolog	Its overexpression in Arabidopsis confers bicarbonate alkaline tolerance (Duan et al., 2017).
*GsBOR2*	Glyso.06G166900	Glyma.06G181900	Boron transporter	Its overexpression in Arabidopsis confers bicarbonate alkaline tolerance (Duan et al., 2018).

Gene ID and ID for *G. max* Homolog were retrieved from the plant genomic resource Phytozome with the *Glycine max* Wm82.a4.v1 and *Glycine soja* v1.1 versions respectively, according to the corresponding information given in the references.

### Cation/H^+^ exchangers

Cation/H^+^ exchangers (CHXs), belonging to the cation/proton antiporter 2 (CPA2) family, are suggested to mediate K^+^, Na^+^, and H^+^ flow (Isayenkov et al., 2020). In 2014, a salt-tolerant gene *Gs/GmCHX1*, the homolog of *AtCHX20*, was identified by whole-genome sequencing of a *G. soja* accession W05, and genotyping-by-sequencing and phenotypic analyses of a recombinant inbred line (RIL) population (Qi et al., 2014). In *G. soja* W05, *GsCHX1* (Glysoja01g005509) encodes a CHX protein with 811 amino acids in length. In *G. max* Williams 82 and C08 (one parent of the RIL population), a Ty1/Copia retrotransposon was inserted into the third exon of *GmCHX1*(Glyma03g32900), resulting in a truncated transcript encoding only 376 residues. In the same year, Guan et al. identified *GmSALT3* (salt tolerance-associated gene on chromosome 3) using the RIL population derived from the salt-tolerant variety Tiefeng 8 and the salt-sensitive variety 85-140 (Guan et al., 2014). In 2016, Do et al. isolated a salt tolerance-related QTL from a Brazilian soybean cultivar FT-Abyara, and named the causal gene as *GmNcl* (Do et al., 2016). Actually, both *GmSALT3* and *GmNcl* are the same gene as *GmCHX1*. Therefore, *GmCHX1/SALT3/Ncl* is a critical determinant of salt tolerance in soybean. *GmCHX1/SALT3/Ncl* contributed to salt tolerance at the seedling stage and conferred improved soybean yield in the field by maintaining a higher seed weight (Do et al., 2016; Liu et al., 2016). However, *GmCHX1/SALT3/Ncl* does not affect the seedling emergence rate or early vigor under salt stress (Liu et al., 2016). Therefore, identifying the key genes improving the seed germination vigor in saline soil is of great importance for salt-tolerant soybean breeding with *GmCHX1/SALT3/Ncl*.

In Guan et al. study, 9 haplotypes of *GmCHX1/SALT3/Ncl*, including 2 salt-tolerant haplotypes and 7 salt-sensitive haplotypes were identified by sequencing 31 soybean landraces and 22 wild soybeans (Guan et al., 2014). In another work, SNPs within the coding region and 2-kb promoter region of *Gs/GmCHX1* were highly conserved in the 12 salt-tolerant accessions but varied among the 11 salt-sensitive accessions (Qi et al., 2014). Recently, 40 different haplotypes were identified in 216 *G. max* and *G. soja* accessions from Korea, China, and Japan (Lee et al., 2018). In summary, the consequence of these SNPs in salt-sensitive accessions is either the dysfunction of Gs/GmCHX1 protein or the deficient levels of *Gs/GmCHX1* transcripts. However, it is still unknown whether there is a regulation of *Gs/GmCHX1* at the post-transcriptional level. Besides, the regulatory mechanism of Gs/GmCHX1 transporting activity also remains obscure.

As a CHX family protein, *GmCHX1/SALT3/Ncl* was found to affect Na^+^, K^+^, and Cl^–^ accumulation under salt stress (**Figure 1A**). Upon salt stress, Na^+^ accumulation in stems and leaves in the salt-tolerant soybean accessions was significantly less than that in the salt-sensitive accessions (Guan et al., 2014; Do et al., 2016; Liu et al., 2016). The salt-tolerant soybean accumulated less K^+^ in leaves and stems than the salt-sensitive line (Do et al., 2016). Interestingly, the salt-tolerant NIL line accumulated less Cl^−^ in the leaves and more Cl^−^ in the roots prior to any difference in Na^+^ (Do et al., 2016; Liu et al., 2016). A recent study showed that *GmCHX1/SALT3/Ncl* expression contributed to net influx and accumulation of Na^+^, K^+^, and Cl^–^ in *Xenopus laevis* oocytes (Qu et al., 2021). In soybean shoots, *GmCHX1/SALT3/Ncl* mediated Na^+^ exclusion by restricting the net xylem loading of Na^+^, and contributed to Cl^-^ exclusion by re-translocating Cl^-^ back into roots *via* the phloem. GmCHX1/SALT3/Ncl protein is localized in the endoplasmic reticulum (ER) in plant cells (Guan et al., 2014; Qi et al., 2014). It is still unknown how the ER-localized GmCHX1/SALT3/Ncl affects the Na^+^ and Cl^-^ exclusion across PM. One possible explanation is that the ER-localized GmCHX1/SALT3/Ncl influences ion gradients across the PM through currently unknown downstream effects. GmCHX1/SALT3/Ncl is also possible to mediate Na^+^ and Cl^-^ exclusion *via* an ER-derived vesicle trafficking system.

Similarly, *GsCHX19.3* is a plasma membrane localized CHX isolated from bicarbonate stress tolerant *G. soja* G07256. Overexpression of *GsCHX19.3* in Arabidopsis resulted in higher K^+^ content under normal growth conditions, and lower Na^+^ accumulation under NaHCO_3_ treatment (Jia et al., 2017), indicating the role of *GsCHX19.3* in both K^+^ uptake and Na^+^ excretion (**Figure 1A**). In Jia et al. study, 34 *GsCHXs* were identified and phylogenetically clustered into five groups (Group I-V). Only five Group IVa members (*GsCHX18.1*, *GsCHX18.2*, *GsCHX19.2*, *GsCHX19.3*, and *GsCHX20*) exhibited enhanced expression at the transcript level under carbonate alkaline stress, indicating the involvement of *GsCHX18/19/20s* in salt-alkaline stress response. Therefore, further studies are needed to identify the key cis-elements and transcription factors that trigger the expression of *GsCHX18/19/20s* under salt-alkaline stress.

### Na^+^/H^+^ antiporters

Na^+^/H^+^ antiporters (NHXs) belong to the cation/proton antiporter 1 (CPA1) family. They play critical roles in the exclusion of cytoplasmic Na^+^ mediated by plasma membrane NHXs and compartmentalization of vacuole Na^+^ driven by vacuole membrane NHXs (Jia et al., 2018). In Arabidopsis, the plasma membrane SOS1 (Salt Overlay Sensitive 1), also called AtNHX7, is the major transporter removing intracellular Na^+^ in exchange for extracellular H^+^. Upon salt stress, SOS3 senses the calcium signal induced by salt stress and interacts with the protein kinase SOS2, thereby activating its kinase activity and recruiting it to the plasma membrane. SOS2 then activates SOS1 Na^+^/H^+^ reverse transport activity to exclude cytoplasmic Na^+^ (Zhu, 2002).

Until now, only one NHX gene *GmsSOS1* from *G. soja* has been functionally reported to modulate salt tolerance (Nie et al., 2015). The *GmsSOS1* gene was cloned from *G. max* and *G. soja* and encoded a plasma membrane NHX protein. Ectopic expression of *GmsSOS1* in the wild type Arabidopsis, as well as the *atsos1-1* mutant deleting 14bp (1330-1343bp) (Shi et al., 2000), improved the salt stress tolerance by decreasing Na^+^ absorption in roots and transportation in shoots (**Figure 1A**). Considering the importance of NHXs in the regulation of cytoplasmic Na^+^ concentration, more attention should be paid to identifying *G. soja* NHX genes and illustrating the main SOS signaling pathway that contributes to salt tolerance.

### Anion channels

In addition to cation toxicity caused by Na^+^, Cl^-^ is the major factor resulting in the anionic toxicity under salt stress. Excessive Cl^-^ in cells disturbs the ${\text{NO}}_{3}^{-}$ uptake (Rosales et al., 2020). Therefore, under salt stress, plants must simultaneously cope with cation and anionic toxicity.

The chloride channels (CLCs) could simultaneously transport Cl^-^ and ${\text{NO}}_{3}^{-}$ (Jentsch and Pusch, 2018). There are eight CLC genes in soybean, among which the tonoplast-localized CLC-c2 showed different expression patterns between the salt-sensitive *G. max* N23674 and salt-tolerant *G. soja* BB52 (Wei et al., 2019). Under salt stress, *GsCLC-c2* overexpression in soybean composite plants increased Cl^-^ and Na^+^ content in roots and reduced Cl^-^ and Na^+^ accumulation in stems and leaves. Furthermore, *GsCLC-c2* overexpression in soybean composite plants also resulted in higher content of ${\text{NO}}_{3}^{-}$ in shoots, without significant changes in K^+^ accumulation (**Figure 1B**). Consequently, *GsCLC-c2* contributes to salt stress tolerance by increasing ${\text{NO}}_{3}^{-}/{\text{Cl}}^{-}$ and K^+^/Na^+^ ratios, especially in shoots (Wei et al., 2019; Liu et al., 2021b). In the *GsCLC-c2* RNAi composite plants, Cl^-^ content was significantly decreased in roots but increased in stems and leaves, while the ${\text{NO}}_{3}^{-}$ content was decreased in roots, stems, and leaves, resulting in the increased ${\text{Cl}}^{-}/{\text{NO}}_{3}^{-}$ ratios (Liu et al., 2021b). In addition, *GsCLC-c2* overexpression in the wild type and *atclc-c* mutant Arabidopsis also led to increased ${\text{Cl}}^{-}/{\text{NO}}_{3}^{-}$ and K^+^/Na^+^ ratios in roots under salt stress. The K^+^/Na^+^ ratio in shoots of *GsCLC-c2* transgenic Arabidopsis was increased under salt stress, however, the ${\text{Cl}}^{-}/{\text{NO}}_{3}^{-}$ ratio was not altered (Liu et al., 2021b). Researchers also suggested that GsCLC-c2 could transport both Cl^-^ and ${\text{NO}}_{3}^{-}$ by using a two-electrode voltage clamp on *Xenopus laevis* oocytes (Wei et al., 2019). Therefore, *GsCLC-c2* plays a key positive role in regulating *G. soja* salt-alkaline tolerance by simultaneously transporting Cl^-^ and ${\text{NO}}_{3}^{-}$ . More importantly, *CLC-c2* is the only gene with non-synonymous changes between *G. max* N23674 and *G. soja* BB52. It is important to investigate the sequence variations of the *CLC-c2* gene in wild and cultivated soybean populations and identify the salt-alkaline tolerant haplotypes.

Besides CLCs, slow anion channels (SLACs) and SLAC homologs (SLAHs) also play critical roles in anion transport (Barbier-Brygoo et al., 2011). Most characterized SLAC/SLAHs are ${\text{NO}}_{3}^{-}$ conductive. An alkaline stress-induced SLAH gene *GsSLAH3*, the homologous gene to *AtSLAH3*, was identified in the bicarbonate (NaHCO_3_) stress tolerant *G. soja* G07256 based on RNA-seq data. Transgenic Arabidopsis over-expressing *GsSLAH3* displayed higher tolerance to ${\text{HCO}}_{3}^{-}$ stress (NaHCO_3_ and KHCO_3_) rather than high pH stress, by increasing ${\text{NO}}_{3}^{-}$ accumulation in shoots (Duan et al., 2017). Consistently, under NaHCO_3_ treatment, the ${\text{NO}}_{3}^{-}$ concentrations in the T-DNA insertion Arabidopsis mutants *atslah3-1* and *atslah3-2* were relatively lower than WT (Zheng et al., 2015) (**Figure 1B**). It is possible that *GsSLAH3* specifically controls the response to carbonate salt-alkaline stress, not high pH stress, by mediating ${\text{NO}}_{3}^{-}$ transport. However, direct evidence supporting the conductivity of GsSLAH3 to ${\text{NO}}_{3}^{-}$ is still missing. Further studies should also analyze the sequence and expression differences of *SLAH* genes between *G. soja* and *G. max*.

### Boron transporters

Boron (B) deficiency decreases ${\text{NO}}_{3}^{-}$ uptake and increases K^+^ leakage across the plasma membrane. Plant B transporters (BORs) belong to the solute carrier (SLC4) family, which is homologous to the human bicarbonate transporter-related protein HsBTR1, and each BOR protein harbors a sodium-coupled bicarbonate transporter domain (Saouros et al., 2021). A recent study reported that a *G. soja* BOR gene *GsBOR2* could improve the tolerance to bicarbonate stress (NaHCO_3_ and KHCO_3_) rather than high pH stress (Duan et al., 2018). Functional complementation showed that *GsBOR2* restored the sensitivity of *Scbor1* mutant yeasts to H_3_BO_3_ treatment, suggesting the involvement of boron and BORs in salt-alkaline stress response (Duan et al., 2018) (**Figure 1C**). However, direct evidence is required to characterize whether BORs affect ${\text{NO}}_{3}^{-}$ uptake and K^+^ leakage under bicarbonate stress.

Till now, only these six *G. soja* genes encoding ion transporters have been functionally characterized to maintain ion balance and alleviate ion toxicity under salt-alkaline stress. Among them, GmCHX1 has been suggested to transport Na^+^, K^+^, and Cl^–^ (Qu et al., 2021), and GsCLC-c2 transported Cl^-^ and ${\text{NO}}_{3}^{-}$ (Wei et al., 2019). Even though the regulation of *GsCHX19.3, GmsSOS1, GsSLAH3*, and *GsBOR2* on the cytoplasmic ion accumulation has been studied through functional complementation in yeast mutants and quantification analyses of ion contents in transgenic plants, direct biochemical evidence is still lacking to verify their transporting properties. Therefore, techniques monitoring ion fluctuation in real-time, for example, non-invasive micro-test technology and two photon-total internal refraction fluorescence microscopy, should be applied to directly verify the transporting capability of ion transporters in *G. soja*. Besides, there are still a few points that need further detailed investigation. Firstly, it is imperative to investigate whether these ion transporter genes could improve soybean yields in the salt-alkaline fields. Secondly, further studies are needed to reveal the regulatory mechanism of the activity of these ion transporters, for example, identifying the protein kinases that trigger their transporting activity and the transcription factors that promote their transcription.

## ROS signaling and scavenging under salt-alkaline stress in wild soybean

**Figure 2:**
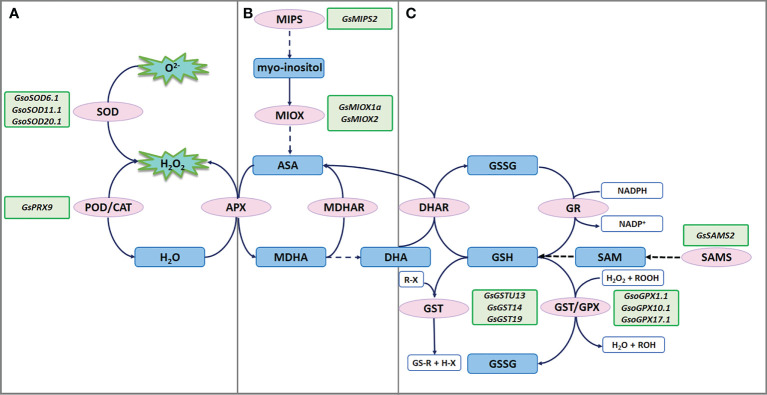
ROS scavenging under salt-alkaline stress in wild soybean. **(A)** SODs and PRXs in enzymatic antioxidant defense system. **(B)** MIPSs and MIOXs responsible for AsA biosynthesis. **(C)** GSTs, GPXs and SAMSs responsible for glutathione mediated nonenzymatic antioxidant defense system.

Under salt-alkaline stress, ion imbalance promotes the generation and accumulation of reactive oxygen species (ROS) in plant cells. ROS play dual roles in plant response to salt-alkaline stress. At low concentrations, it can serve as a signaling molecule, which activates downstream signal transduction under salt-alkaline stress. However, ROS accumulation at high concentrations leads to oxidative damage or apoptotic death and damages plant cells (Liu et al., 2021c). Plants have developed enzymatic and nonenzymatic antioxidant defense systems to protect against oxidative damage. For now, a serial of genes involved in ROS signaling and scavenging have been transcriptionally and functionally characterized to regulate salt-alkaline stress tolerance in *G. soja* (**Table 2**; **Figure 2**).

**Table 2:** Genes involved in ROS scavenging under salt-alkaline stress in wild soybean.

Gene name	Gene ID	ID for *G. max* Homolog	Gene description	Function description
*GsoSOD6.1*	Glyso.06G132100	Glyma.06G144500	Superoxide dismutase	Its expression responds to salt stress (Aleem et al., 2022).
*GsoSOD11.1*	Glyso.11G155700	Glyma.11G192700	Superoxide dismutase	Its expression responds to salt stress (Aleem et al., 2022).
*GsoSOD20.1*	Glyso.20G036200	Glyma.20G050800	Superoxide dismutase	Its expression responds to salt stress (Aleem et al., 2022).
*GsoGPX1.1*	Glyso.01G178300	Glyma.01G219400	Glutathione peroxidase	Its expression responds to salt stress (Aleem et al., 2022).
*GsoGPX10.1*	Glyso.10G022800	Glyma.10G024600	Glutathione peroxidase	Its expression responds to salt stress (Aleem et al., 2022).
*GsoGPX17.1*	Glyso.17G189500	Glyma.17G223900	Glutathione peroxidase	Its expression responds to salt stress (Aleem et al., 2022).
*GsPRX9*	Glyso.14G062700	Glyma.14G070800	Secretory peroxidase	Its overexpression in soybean composite plants confers salt tolerance (Jin et al., 2019).
*GsSAMS2*	Glyso.17G036200	Glyma.17G039100	S-Adenosyl-L-Methionine synthetase	Its overexpression in alfalfa confers salt tolerance (Hua et al., 2012).
*GsGST*	Glyso.01G081200	Glyma.01G106000	Tau class glutathione S-transferase	Its overexpression in tobacco confers salt tolerance (Ji et al., 2010).
*GsGST13/GSTU13*	Glyso.03G056100	Glyma.03G073702	Tau class glutathione S-transferase	Its overexpression in alfalfa confers salt and bicarbonate alkaline tolerance (Wu et al., 2014; Jia et al., 2015).
*GsGST14*	Glyso.05G135100	Glyma.05G161600	Tau class glutathione S-transferase	Its overexpression in alfalfa confers salt and bicarbonate alkaline tolerance (Wang et al., 2012b; Lin et al., 2013).
*GsGST19*	Glyso.02G157600	Glyma.02G187200	Tau class glutathione S-transferase	Its overexpression in alfalfa confers salt and bicarbonate alkaline tolerance (Wang et al., 2012b).
*GsGSTL1*	Glyso.03G139400	Glyma.03G176300	Lambda class glutathione S-transferase	Its overexpression in tobacco BY-2 cells and Arabidopsis confers salt tolerance (Chan, 2014)
*GsMIOX1a*	Glyso.08G182700	Glyma.08G199300	Myo-inositol oxygenase	Its overexpression in Arabidopsis confers bicarbonate alkaline tolerance (Chen et al., 2015a).
*GsMIPS2*	Glyso.18G020300	Glyma.18G022100	L-myo-inositol-1-phosphate synthase	Its overexpression in Arabidopsis confers bicarbonate alkaline tolerance (Chen et al., 2015b).

Gene ID and ID for *G. max* Homolog were retrieved from the plant genomic resource Phytozome with the *Glycine max* Wm82.a4.v1 and *Glycine soja* v1.1 versions respectively, according to the corresponding information given in the references.

### ROS-activated signaling under salt-alkaline stress in wild soybean

Previous studies have demonstrated the positive role of ROS in salt-alkaline stress response by activating the downstream signaling. Exogenous H_2_O_2_ application could improve salt tolerance in rice and maize (Uchida et al., 2002; Azevedo Neto et al., 2005). ROS-production deficiency *Arabidopsis* mutants *atrbohd* and *atrbohf* are sensitive to salt stress (Ben Rejeb et al., 2015). Recently, it has been reported that H_2_O_2_ could sulfenylate TSB1 (tryptophan synthase β subunit 1) to increase ABA accumulation, thus increasing salt stress tolerance in Arabidopsis (Liu Y. L. et al., 2022). A recent study in *G. max* reported that a ROS-involving positive feed-forward loop acted as a signal amplifier in salt stress response (Li et al., 2019). Overexpression of a NAC transcription factor *GmSIN1* (SALT INDUCED NAC1) in soybean promoted root growth and salt tolerance and increased yield under salt stress. *GmSIN1* upregulated expression of *GmRbohB* (Respiratory burst oxidase homolog B) genes to generate ROS, and ROS further induced *GmSIN1* expression under salt stress (Li et al., 2019). Even though researchers have unraveled the ROS-mediated regulatory mechanism in plant response to salt stress, its role is rarely reported in *G. soja*. A previous study reported that a methionine sulfoxide reductase B (MSRB) gene in *G. soja GsMSRB5a* could regulate alkaline stress tolerance by modifying the expression of ROS signaling genes (Sun et al., 2016). Therefore, more studies are needed to clarify the positive regulatory mechanism mediated by ROS on salt-alkaline tolerance in *G. soja*.

### Antioxidant enzymes

Enzymes that detoxify ROS include superoxide dismutases (SODs), catalases (CATs), peroxidases (PODs), glutathione peroxidases (GPXs), ascorbate peroxidases (APXs), monodehydroascorbate reductases (MDHARs), dehydroascorbate reductases (DHARs), and glutathione reductases (GRs). Among them, SODs serve as the front line of antioxidant defense by catalyzing ${\text{O}}_{2}^{-}$ radicals into H_2_O_2_ and molecular oxygen (O_2_). A recent study showed that *G. soja* W05 had a total of 13 SOD genes (*GsoSODs*), and the expression of three *GsoSODs* (*GsoSOD6.1, GsoSOD11.1*, and *GsoSOD20.1*) has been found to respond to salt stress *via* qRT-PCR analyses (Aleem et al., 2022). Under 250mM NaCl treatment, *GsoSOD11.1* showed continuously up-regulated expression (**Figure 2A**). The increase in *GsoSODs* expression will help to remove ${\text{O}}_{2}^{-}$ radicals.

H_2_O_2_ is subsequently removed by CATs and PODs. In plants, PODs are divided into three classes: class I (Ascorbate peroxidase, APXs), class II (lignin peroxidases), and class III (secretory peroxidases, PRXs). Class III PRXs are bifunctional enzymes that could scavenge ROS and also produce ROS. A recent study suggested that overexpression of salt stress-induced *G. soja* PRX gene *GsPRX9* in soybean composite plants improved salt tolerance by increasing both POD and SOD activity, and glutathione levels (Jin et al., 2019) (**Figure 2A**). The coding sequence of *GsPRX9* is identical to that of *GmPRX9*. Interestingly, in *G. max*, *GmPRX9* expression showed a significantly higher increase in the salt-tolerant varieties than the salt-sensitive varieties after salt treatment. These findings imply that the expression level of *PRX9* is related to the salt tolerance of soybean. Therefore, it will be interesting to check the sequences of the *PRX9* promoter in the soybean natural population to identify the haplotype that confers higher expression levels of *PRX9* and higher salt tolerance.

Even though the expression response and genetic function of *CAT* and *APX* genes under salt-alkaline stress have not been reported in *G. soja*, and CATs and APXs indeed play crucial roles in ROS scavenging in wild soybean response to salt-alkaline stress. For example, compared with a cultivated soybean ZH13, the wild soybean BB52 grown in coastal saline land in the Yellow River Delta displayed higher CAT and APX activities under salt stress (Chen et al., 2013). Therefore, it is essential to identify *CATs* and *APXs* genes in *G. soja* that could improve the tolerance to salt-alkaline stress.

### Ascorbic acid

Ascorbic acid (AsA) contributes to abiotic stress tolerance by scavenging ROS through the AsA-GSH cycle. Myo-inositol is one of the main precursors of AsA biosynthesis, while L-myo-inositol-1-phosphate synthases (MIPSs) are rate-limiting enzymes in myo-inositol biosynthesis (Donahue et al., 2010). There are three *MIPS* genes in soybean. According to the public RNA-seq data of the salt-alkaline tolerant *G. soja* 07256, *GsMIPS2* expression was dramatically increased by about 20 folds at 3 h treated with 50 mM NaHCO_3_, which was further verified *via* qRT-PCR analyses. Transcript levels of *GsMIPS3* was decreased, while *GsMIPS1* expression was not changed by NaHCO_3_ treatment. Consistently, *GsMIPS2* overexpression in Arabidopsis conferred increased tolerance to NaHCO_3_ and NaCl stresses, and the T-DNA insertion Arabidopsis mutant *atmips2* displayed reduced tolerance (Chen et al., 2015b; Nisa, 2016) (**Figure 2B**).

Myo-inositol oxygenases (MIOXs) catalyze the conversion of myo-inositol into D-glucuronic acid (D-GlcUA), which is finally oxidized into AsA (Endres and Tenhaken, 2009). A previous study reported that there were 5 MIOX genes in wild soybean and *GsMIOX1a* expression displayed a noticeable increase at 6 h treated with 50 mM NaHCO_3_ (Chen et al., 2015a). Interestingly, expression of *GsMIOX1a* was dramatically induced by NaHCO_3_ treatment in *G. soja* accessions (G07256 and G50109), but not in *G. max* accessions (Suinong 28 and Hefeng 55). This difference indicates that *GsMIOX1a* might be a key factor for *G. soja* response to NaHCO_3_ stress. Genetic evidence suggests that *GsMIOX1a* overexpression in Arabidopsis improved POD activity and increased the bicarbonate alkaline stress tolerance (Chen et al., 2015a) (**Figure 2B**). Taken together, it is absolute that AsA biosynthesis might contribute to soybean tolerance to salt-alkaline stress, possibly *via* the AsA-GSH cycle.

### Glutathione

Glutathione is one important component of the nonenzymatic antioxidant system, having two forms reduced glutathione (GSH) and oxidized glutathione (GSSG). The glutathione peroxidases (GPXs) catalyze the oxidation of GSH to produce GSSG, reducing H_2_O_2_ or organic hydroperoxide to H_2_O and alcohol, which will protect cells against ROS-mediated oxidative damage. A recent study showed that *G. soja* W05 has 13 GPX (*GsoGPXs*) genes, three of which were responsive to salt stress (Aleem et al., 2022). Under 250mM NaCl treatment, *GsoGPX10.1* showed continuously up-regulated expression (**Figure 2C**). Even though no genetic evidence supporting the function of *GsoGPXs* in regulating salt tolerance has been given, it is easy to speculate that the increase in *GsoGPXs* transcript levels will facilitate the removal of H_2_O_2_ or organic hydroperoxide.

S-Adenosyl-L-Methionine Synthetases (SAMSs) catalyze the synthesis of S-Adenosyl-L-Methionine (SAM), which generates glutathione (GSH) through sulfur transfer. Hence, the activity of SAMSs is closely related to the GSH content in plant cells (Hasanuzzaman et al., 2017). It has been reported that ectopic expression of a *G. soja* SAMS gene *GsSAMS2* driven by a stress-inducible promoter RD29A conferred salt tolerance in *Medicago sativa* (Hua et al., 2012) (**Figure 2C**). It will be an easy way to improve the GSH content by overexpressing *SAMS* genes, which can help plants to cope with ROS damage.

The glutathione S-transferases (GSTs) catalyze the conjugation of GSH to the electrophilic groups of a large variety of hydrophobic molecules to detoxify cells. Under abiotic stress, GSTs detoxify the lipid hydroperoxides generated in membrane peroxidation. GSTs could be grouped into six classes, and most GSTs in plants belong to the phi (GSTF) and tau (GSTU) classes. Several studies have revealed the regulatory function of GSTUs in *G. soja* response to salt-alkaline stress (**Figure 2C**). A previous study isolated a GSTU gene *GsGST* from the cDNA library of salt-treated *G. soja* 50109 seedlings, and overexpression of *GsGST* in *Nicotiana tabacum* significantly improved the salt and drought tolerance (Ji et al., 2010). Furthermore, three *G. soja* GSTUs *GsGST13* (also named *GsGSTU13*), *GsGST14* and *GsGST19* were found to contribute to higher tolerance under both salt (NaCl), and bicarbonate (NaHCO_3_) stresses (Wang et al., 2012a; Wang et al., 2012b; Jia et al., 2015). In another study, overexpression of *GsGSTU24* and *GsGSTU42* in soybean hairy root composite plants and transgenic Arabidopsis seedlings significantly enhanced antioxidant ability to detoxify ROS damage under submergence stress (Li et al., 2020). Experimental data are needed to determine whether *GsGSTU24* and *GsGSTU42* regulate salt-alkaline tolerance. In addition, a *lambda* class GST gene *GSTL1* was suggested to be induced by salt stress by over 7 folds in *G. soja* W05, compared to less than 4 folds in *G. max* C08 (Chan, 2014). *GSTL1* expression in tobacco BY-2 cells and Arabidopsis increased survival rates under salt stress by reducing ROS accumulation. In summary, *GST* genes are important candidates for improving salt-alkaline tolerance *via* transgene technology.

### Flavonoids

Flavonoids are a group of secondary metabolites derived from the phenylpropanoid pathway, including flavonol, flavanol, flavone, isoflavone, flavanone, proanthocyanidins, and anthocyanin (Liu et al., 2021d). Current studies have demonstrated that flavonoids, as antioxidants, are closely associated with salt-alkaline stress tolerance in soybean. For example, the knockout of a flavone synthase (FNS) gene *GmFNSII* in soybean hairy roots reduced the salt tolerance. *GmMYB183* activated the expression of *GmCYP81E11* (Cytochrome P450 monooxygenase) and increased the accumulation of monohydroxy B-ring flavonoids, which negatively regulates soybean tolerance to salinity (Pi et al., 2019). Another MYB transcription factor *GmMYB173* directly activated the transcription of *GmCHS5* and contributed to salt tolerance by enhancing the accumulation of dihydroxy B-ring flavonoids (Pi et al., 2018). Recently, researchers have found that soybean HSFB2b, a class B heat shock factor, improved salt tolerance by promoting flavonoid biosynthesis (Bian et al., 2020). Four haplotypes of HSFB2b in its promoter region were identified among the population consisting of 38 wild soybean and 113 cultivated soybean accessions, and haplotypes II and III from salt-tolerant wild soybean display higher promoter activity under salt stress. The distribution frequency of haplotype III, which showed the highest promoter activity, in cultivated soybeans was very low. Therefore, it will be an effective way to introduce the haplotype III promoter of *GsHSFB2b* into cultivated soybean to breed salt-tolerant cultivars.

ROS balance is regulated by generation, removal, and transportation. Currently, most studies in *G. soja* focused on the regulation of ROS scavenging under salt-alkaline stress. Genes responsible for ROS generation in *G. soja* have not been reported until now. Furthermore, little is known about the positive role of ROS-activated signaling pathways in *G. soja*. Therefore, future studies regarding ROS-mediated regulation on salt-alkaline response should focus on two points. Firstly, respiratory burst oxidase homologues (Rboh) genes in *G. soja* should be systematically investigated to uncover the mechanism driving the reduction of ROS generation under salt-alkaline stress. Secondly, identification of target genes that are directly activated by ROS at low concentrations is urgently needed to understand the positive role of ROS by serving as signaling molecules under salt-alkaline stress.

## Protein kinases involved in salt-alkaline stress response in wild soybean

**Figure 3:**
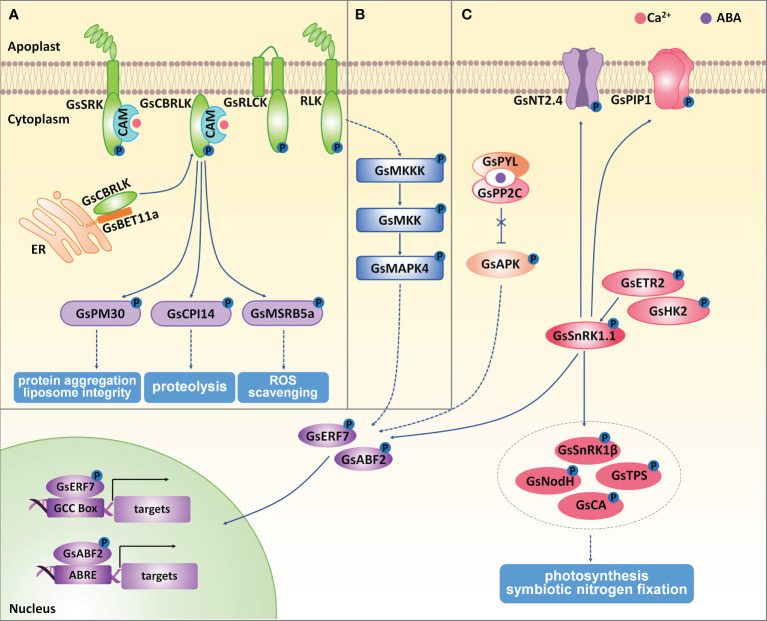
The regulation of protein kinases on wild soybean response to salt-alkaline stress. **(A)** Receptor like kinases, **(B)** MAPK kinases, **(C)** SnRK kinases mediated stress response in *G. soja* under salt-alkaline stress. ER, endoplasmic reticulum. Solid lines represent direct physical interaction, and dotted lines represent indirect interaction or results. Arrows represent positive effects, and lines ending with a short bar indicate negative effects.

Protein kinases (PKs) transfer phosphate groups from adenosine triphosphate (ATP) to serine, threonine, or tyrosine residues of substrate proteins, thereby directly affect substrates’ function by modifying their activity, 3D structure, subcellular localization, or protein stability. The PK superfamily is one of the largest and most highly conserved families in plants. There are 2166 putative PK genes in soybean genome, representing 4.67% of all protein-coding genes (Liu et al., 2015). PKs could be classified into different families based on sequence similarity and domain structure within and outside the catalytic domains. In *G. soja*, several kinase genes from the RLKs (receptor like kinases), MAPKs (mitogen-activated protein kinases), and SnRKs (sucrose non-fermenting1-related protein kinases) families have been reported to play important roles in signal transduction under salt-alkaline stress (**Table 3**; **Figure 3**).

**Table 3:** The genes encoding protein kinases involved in regulating salt-alkaline stress response in wild soybean.

Gene name	Gene ID	ID for *G. max* Homolog	Gene description	Function description
*GsSRK*	Glyso.06G220200	Glyma.06G255900	Lectin receptor like kinase	Its overexpression in Arabidopsis and alfalfa confers salt tolerance (Sun et al., 2013b; Sun et al., 2018).
*GsRLCK*	Glyso.17G108900	Glyma.17G117800	Receptor like cytoplasmic kinase	Its overexpression in Arabidopsis confers salt and drought tolerance (Sun et al., 2013a).
*GsCBRLK/CRCK1d*	Glyso.18G058500	Glyma.18G064100	Receptor like cytoplasmic kinase	Its overexpression in soybean, alfalfa, rice and Arabidopsis confers salt and bicarbonate alkaline tolerance (Bai et al., 2013; Zhao et al., 2014; Ji et al., 2015; Cai et al., 2020).
*GsCRCK1a*	Glyso.02G185500	Glyma.02G224000	Receptor like cytoplasmic kinase	Its expression is induced by salt and bicarbonate alkaline stresses (Sun et al., 2016).
*GsCRCK1b*	Glyso.11G182100	Glyma.11G178300	Receptor like cytoplasmic kinase	Its expression is induced by salt and bicarbonate alkaline stresses (Feng et al., 2020).
*GsCRCK1c*	Glyso.14G151000	Glyma.14G190700	Receptor like cytoplasmic kinase	Its expression is induced by salt and bicarbonate alkaline stresses (Zhu et al., 2012a).
*GsSnRK1.1*	Glyso.13G037400	Glyma.13G060400	SnRK1 kinase	Its overexpression in soybean composite plants confers salt tolerance (Feng et al., 2020).
*GsSnRK1.2*	Glyso.18G205100	Glyma.18G262900	SnRK1 kinase	Its expression is induced by salt and bicarbonate alkaline stresses (Chen et al., 2021a).
*GsAPK*	Glyso.01G164500	Glyma.01G204200	SnRK2b kinase	Its overexpression in Arabidopsis confers salt tolerance (Yang et al., 2012).
*GsMAPK4*	Glyso.01G180800	Glyma.01G222000	MAPK kinase	Its overexpression in soybean confers salt tolerance (Qiu et al., 2019).

Gene ID and ID for *G. max* Homolog were retrieved from the plant genomic resource Phytozome with the *Glycine max* Wm82.a4.v1 and *Glycine soja* v1.1 versions respectively, according to the corresponding information given in the references.

### Receptor like kinases

RLKs consist of an extracellular domain to receive signal molecules, a transmembrane domain and a cytoplasmic kinase catalytic domain. RLKs could be further classified into different groups, for example, LRR-RLK (leucine-rich repeat RLK), LecRLK (Lectin RLK), RLCK (cytoplasmic RLK). In soybean, 1418 genes were identified as RLKs (Liu et al., 2015), including 467 LRR-RLKs (Zhou et al., 2016) and 185 LecRLKs (Liu et al., 2018). Until now, only a few *G. soja* RLKs were reported to involve in salt-alkaline stress (**Figure 3A**).

*GsSRK* encodes a LecRLK protein from *G. soja*, whose extracellular domain consists of an N-terminal signal peptide, a G-type lectin domain, an S-locus-glycop domain, and a PAN-AP domain (Sun et al., 2013b). *GsSRK* expression was greatly and rapidly induced by ABA, salt, and drought stresses. *GsSRK* overexpression in Arabidopsis enhanced salt stress tolerance and promoted seed yields under salt stress (Sun et al., 2013b). Furthermore, ectopic expression of the full-length (*GsSRK-f*) and truncated *GsSRK* deleting the G-type lectin domain (*GsSRK-t*) in alfalfa both increased salt tolerance by improving the capacity in ion homeostasis, ROS scavenging, and osmotic regulation. Interestingly, *GsSRK-t* transgenic lines were more tolerant to salt stress than *GsSRK-f* lines, showing more branches and higher fresh weight under salt stress. However, no difference in the physiological indices under salt stress was detected between *GsSRK-f* and *GsSRK-t* transgenic lines (Sun et al., 2018). Interestingly, overexpression of *GsSRK-t*, but not *GsSRK-f*, caused more branches and shorter shoots, indicating a potential role of the extracellular G-type lectin domain in regulating plant architecture. Therefore, *GsSRK* is an ideal target to improve yield under salt stress.

Current studies also demonstrated the important roles of *G. soja* RLCKs in regulating salt-alkaline stress tolerance. *GsRLCK* encodes a plasma membrane localized RLCK, containing a conserved catalytic domain and two transmembrane domains at the N-terminus (Sun et al., 2013a). The expression of *GsRLCK* was induced by ABA, salt, alkaline, and drought stresses. *GsRLCK* overexpression in Arabidopsis increased salt and drought tolerance by promoting the expression of salt responsive marker genes. Furthermore, a group of calcium/calmodulin-binding receptor-like cytoplasmic kinases (CRCKs) in *G. soja* (GsCRCK1a-d) were reported to function in salt-alkaline stress response. GsCRCK1s, without any extracellular or transmembrane domain, contained only intracellular catalytic domains whose amino acid sequences are highly conserved. The N-terminal sequences of GsCRCK1s were quite variable, named the variable domain. This domain has been demonstrated to fulfill a crucial role in mediating the protein-protein interaction and subcellular localization (Sun et al., 2016; Sun et al., 2019; Sun et al., 2021). It has been suggested that GsCRCK1d, also named GsCBRLK (*G. soja* calcium/calmodulin-binding receptor-like kinase), localized on plasma membrane in plant cells. The N-terminal variable domain was responsible for its plasma membrane localization (Yang et al., 2010; Sun et al., 2021). *GsCBRLK* was initially characterized to confer salt and carbonate alkaline tolerance by overexpressing in Arabidopsis (Yang et al., 2010; Sun et al., 2016). Further studies verified its positive regulation on soybean (Ji et al., 2015), alfalfa (Bai et al., 2013; Zhao et al., 2014), and rice (Cai et al., 2020) tolerance to both salt and carbonate alkaline stresses.

Current studies have identified the interacting partners of GsCBRLK and illustrated the functional mechanism and signal transduction mediated by GsCBRLK in salt-alkaline stress response (Yang et al., 2010; Sun et al., 2014; Sun et al., 2016; Sun et al., 2019; Sun et al., 2021). Upon salt-alkaline stress, cytoplasmic free Ca^2+^ ([Ca^2+^]cyt) was rapidly accumulated in a short time, and this [Ca^2+^]cyt oscillation was sensed by calmodulins (CaMs). The Ca^2+^/CaM complex activated GsCBRLK kinase activity by directly interacting with CaM and the CaM binding domain within GsCBRLK (Yang et al., 2010). Recently, a group of BET1-like soluble NSF attachment protein receptor (SNARE) proteins (GsBET11a/b/c and GsBET12a/b) were suggested to mediate the plasma membrane localization of GsCBRLK by directly interacting with the N-terminal variable domain of GsCBRLK, and contributed to salt stress tolerance (Sun et al., 2021). For now, three types of GsCBRLK interacting partners, including a cysteine proteinase inhibitor GsCPI14 (Sun et al., 2014), several group 3A late embryogenesis abundant (LEA) proteins (Sun et al., 2019), methionine sulfoxide reductase B (MSRB) subfamily proteins (Sun et al., 2016), have been identified as potential downstream substrates of GsCBRLK. Among them, *GsCPI14* was suggested to regulate the proteolysis process and confer the tolerance to carbonate alkaline stress. The group 3A LEA gene *GsPM30* was found to improve salt tolerance, possibly by preventing protein aggregation and maintaining liposome integrity. *GsMSRB5a* overexpression in Arabidopsis could increase the tolerance to carbonate alkaline stress by enhancing the ROS scavenging capacity. Even though no biochemical evidence has been given to show the direct phosphorylation of GsCBRLK on these proteins, there are two pieces of evidence supporting that GsCBRLK might work upstream of GsMSRBs. Firstly, *GsCBRLK* overexpression in Arabidopsis, soybean and alfalfa conferred tolerance to both salt and carbonate alkaline stresses, while *GsMSRB5a* only activated carbonate alkaline stress responses when overexpressed in Arabidopsis. Secondly, the total MSR enzyme activity was obviously increased in both *GsCBRLK* and *GsMSRB5a* transgenic lines. Therefore, future studies are needed to determine the phosphorylation of GsCBRLK on its substrates. Moreover, it will be helpful to identify the upstream regulators that directly interact with and activate GsCBRLK.

### MAPK kinases

The MAPK kinase cascade has been well documented to play important roles in abiotic stress response in plants (Lin et al., 2021). A MAPK module generally comprises three kinases: a MAPKKK (MAPK kinase), a MKK (MAPK kinase), and a MAPK. In this module, MAPKKKs phosphorylate and activate MKKs, and MKKs then phosphorylate and activate MAPKs. In soybean, there are 38 MAPKs, 11 MKKs, and 150 MKKKs. According to the amino acid sequences, these 38 MAPKs could be clustered into four groups (Group A-D) (Neupane et al., 2013). Even though a number of researches have strongly demonstrated the important roles of MAPK pathway in abiotic stress response in Arabidopsis and rice, only two MAPK kinases in *G. max*, *GMK1/GmMPK6* (Group D) (Im et al., 2012; Im et al., 2021) and *GmMMK1* (Group B) (Liao et al., 2021), have been functionally characterized to regulate salt tolerance. By association analysis of *GmMKK1* sequence variations and salt tolerance in the natural soybean population, researchers showed that the sequence variation in *GmMKK1* promoter was the main reason for the functional differences of *GmMKK1* in the natural population in terms of the germination rates under salt treatment. In *G. soja*, *GsMAPK4* (Group B) was the only MAPK kinase reported to participate in salt stress response. *GsMAPK4* expression was up-regulated under salt stress, and overexpression of *GsMAPK4* in *G. max* significantly improved the tolerance to salt stress (Qiu et al., 2019) (**Figure 3B**). However, the upstream MKKs and MKKKs of *GsMAPK4* are still unknown. Therefore, it is vital to identify the core MAPK components in soybean response to salt-alkaline stress.

### SnRK kinases

In plants, SnRK family kinases are classified into SnRK1, SnRK2, and SnRK3 subfamilies (Wang et al., 2019b). In *G. soja*, there are four SnRK1 genes named *GsSnRK1.1-1.4* (Chen et al., 2021b). Among them, the expression of *GsSnRK1.1* and *GsSnRK1.2* were induced by both salt (NaCl) and bicarbonate alkaline (NaHCO_3_) stresses, while *GsSnRK1.3* and *GsSnRK1.4* were not affected by either salt or bicarbonate alkaline stress. By using the *Agrobacterium rhizogenes*-mediated transformation of soybean hairy roots, *GsSnRK1* (identical to *GsSnRK1.1*) overexpression contributed to higher salt tolerance (Feng et al., 2020). Researchers have identified the putative substrates of GsSnRK1 by using the yeast two-hybrid assays (Song et al., 2019) and the quantitative phosphoproteomics technology (Li et al., 2022) (**Figure 3C**). Phosphorylation assays found that GsSnRK1 phosphorylated plasma membrane-localized GsNT2.4 (Nitrate transporter 2.4) and GsPIP1 (Plasma membrane intrinsic protein 1), which might be involved in the nitrate and water uptake under salt-stress. GsSnRK1β (SnRK1 beta subunit), GsNodH (Sulfotransferase NodH), GsTPS (Alpha, alpha-trehalose‐phosphate synthase) and GsCA (Carbonic anhydrase) were also phosphorylated by GsSnRK1, suggesting the possible role of GsSnRK1 in the regulation of photosynthesis and symbiotic nitrogen fixation. Furthermore, two transcription factors GsERF7 (ethylene-responsive factor 7) and GsABF2 (ABRE‐binding bZIP factor 2) were also demonstrated to be phosphorylation substrates of GsSnRK1 (Li et al., 2022). The phosphorylation of GsERF7 by GsSnRK1 could facilitate its translocation from the cytoplasm to the nucleus and enhance its transactivation activity. GsERF7 could bind the GCC cis-acting element and up-regulate the expression levels of abiotic stress-responsive and hormone synthetic genes. *GsERF7* overexpression, especially co-overexpression with GsSnRK1, could significantly improve soybean tolerance to salt and bicarbonate alkaline stresses (Feng et al., 2020). In addition, two phytohormone‐related histidine kinases GsHK2 (Histidine Kinase 2-like) and GsETR2 (Ethylene receptor 2-like), whose C‐terminal kinase domain interacted with GsSnRK1, were suggested to phosphorylate GsSnRK1.1 in plant cells (Song et al., 2019). In the future, more genetic and physiological experiments are needed to clearly show the regulatory function of these GsSnRK1 interacting partners in salt-alkaline stress response.

SnRK2 kinases have been well-demonstrated as core components of ABA-mediated stress signaling. An SnRK2b-type kinase from *G. soja*, GsAPK, was found to contribute to salt stress tolerance when ectopically expressed in Arabidopsis (Yang et al., 2012). GsAPK exhibited ABA-activated and Ca^2+^-independent kinase activity (**Figure 3C**). The first abscisic acid-activated and Ca^2+^-independent protein kinase (AAPK) was identified in the fava bean (Li and Assmann, 1996). AtSnRK2.6/OST1 (Open stomatal 1) is the homologous protein to fava bean AAPK, and also displayed ABA-activated and Ca^2+^-independent kinase activity (Maszkowska et al., 2021). It is possible that the negative regulator PP2Cs was inhibited upon ABA treatment, thereby resulting in the successful activation of GsAPK. As expected, *GsAPK* overexpression Arabidopsis displayed higher ABA insensitivity, suggesting that *GsAPK* regulated salt tolerance *via* the ABA dependent signaling pathway. In Arabidopsis, SnRK2.2, SnRK2.3, and SnRK2.6 acted as redundant ABA-activated SnRK2s and functioned together in ABA-mediated stress response (Nakashima et al., 2009). There are 22 SnRK2 kinases in soybean (Zhao et al., 2017). Therefore, more work is needed for the functional characterization of soybean *SnRK2* genes in salt-alkaline stress.

In summary, current research has characterized the function of several protein kinase genes, including *GsSRK*, *GsRLCK*, *GsCRCK1d*, *GsSnRK1*, *GsAPK2*, and *GsMPK4*, in regulating salt-alkaline tolerance. Several studies have identified interacting partners of GsCRCK1d and GsSnRK1 to illustrate their regulatory role in salt-alkaline stress signal transduction. However, more work is needed to depict the regulatory network in which PKs occupy a core connecting role. Based on the current reports in *G. soja*, some interesting hypotheses are needed to verify. For example, it is an interesting work to determine whether these functionally characterized PKs could phosphorylate the ion transporters or antioxidant enzymes reported in *G. soja* to mediate the salt-alkaline stress responses. Besides, a multi-year filed trial is needed to evaluate the potential application of these *G. soja* PK genes in improving soybean yields on salt-alkaline fields.

## Transcription factors regulating salt-alkaline stress response in wild soybean

**Figure 4:**
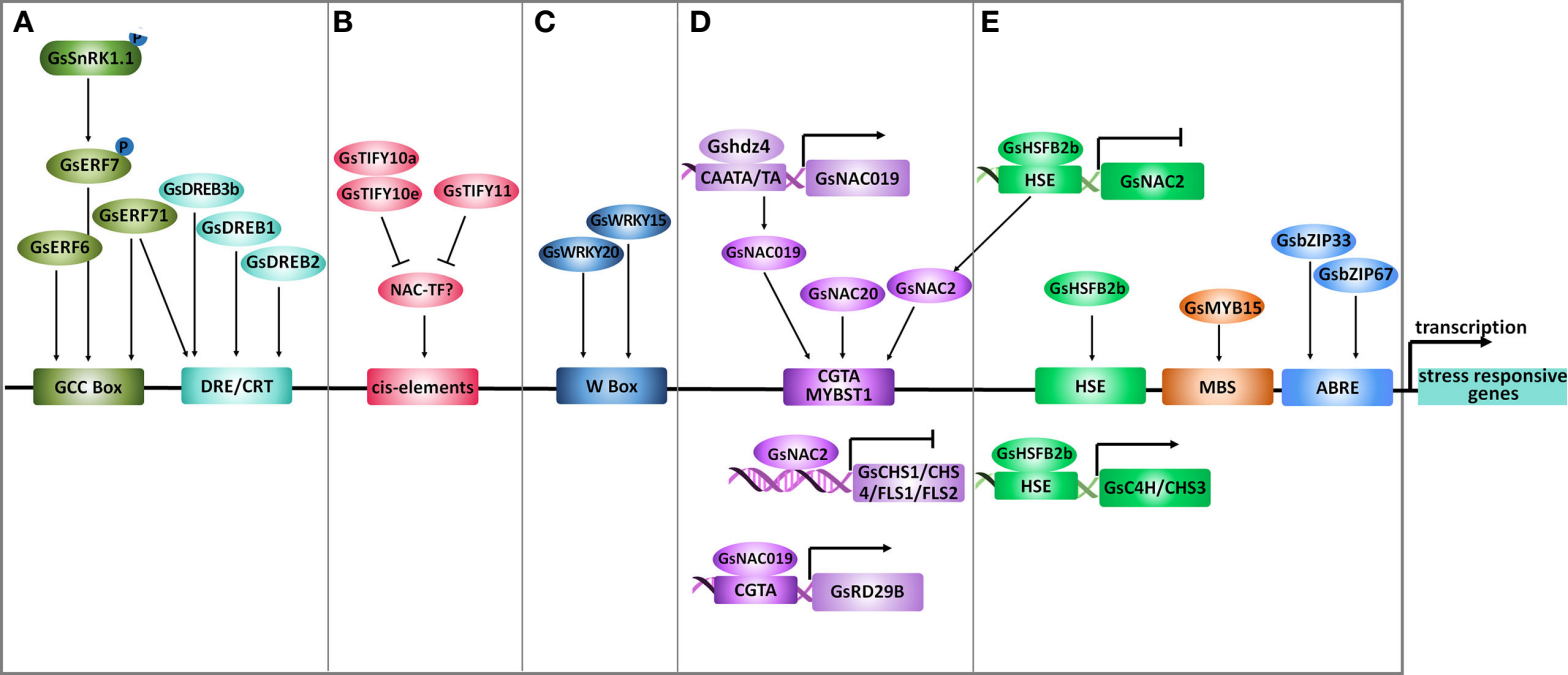
Transcriptional regulation under salt-alkaline stress in wild soybean. **(A)** AP2/ERFs, **(B)** TIFYs, **(C)** WRKYs, **(D)** NACs and **(E)** other transcription factors mediating the transcriptional response of *G. soja* to salt-alkaline stress. Arrows represent positive effects, and lines ending with a short bar indicate negative effects.

Transcription factors (TFs) are key regulators of transcriptional changes and core components of signal transduction in plant response to abiotic stress. Researchers have identified a great deal of TFs in soybean genome, which belong to different families, such as APETALA2/ethylene-responsive factors (AP2/ERF), TIFY, WRKY, homeodomain leucine zipper (HD-Zip), basic helix-loop-helix (bHLH), Cys2/His2-type zinc finger (C2H2), NAC (no apical meristem (NAM), *Arabidopsis thaliana* transcription activation factor (ATAF1/2) and cup-shaped cotyledon (CUC2)). Up to now, genetic evidence has been reported to show the function of AP2/ERFs, TIFYs, WRKYs, and NACs in *G. soja* response to salt-alkaline stress (**Table 4**; **Figure 4**).

**Table 4:** The genes encoding transcription factors involved in regulating salt-alkaline stress response in wild soybean.

Gene name	Gene ID	ID for *G. max* Homologs	Gene description	Function description
*GsERF6*	Glyso.05G057200	Glyma.05G063500	ERF subfamily	Its overexpression in Arabidopsis confers bicarbonate alkaline tolerance (Yu et al., 2016a).
*GsERF71*	Glyso.02G015000	Glyma.02G016100	ERF subfamily	Its overexpression in Arabidopsis confers bicarbonate alkaline tolerance (Yu et al., 2017).
*GsERF7*	Glyso.16G011200	Glyma.16G012600	ERF subfamily	Its overexpression in soybean transgenic hairy roots confers salt and bicarbonate alkaline tolerance (Feng et al., 2020).
*GsDREB3b*	Glyso.04G093800	Glyma.04G103900	DREB subfamily	Its overexpression in soybean transgenic hairy roots confers salt tolerance (Hou et al., 2022).
*GsDREB1*	Glyso.01G150800	Glyma.01G188600	DREB subfamily	(Hu et al., 2007)
*GsDREB2*	Glyso.06G038100	Glyma.06G042100	DREB subfamily	Overexpression of GsDREB2 deleting the negative regulatory domain in Arabidopsis confers salt tolerance (Cai et al., 2018).
*GsTIFY10a*	Glyso.15G161400	Glyma.15G179600	JAZ subfamily	Its overexpression in Arabidopsis and alfalfa confers bicarbonate alkaline tolerance (Zhu et al., 2011; Zhu et al., 2014).
*GsTIFY10e/JAZ2*	Glyso.01G164700	Glyma.01G204400	JAZ subfamily	Its overexpression in Arabidopsis and soybean confers bicarbonate alkaline tolerance (Zhu et al., 2012a; Zhao et al., 2020).
*GsTIFY11b*	Glyso.16G008900	Glyma.16G010000	JAZ subfamily	Its overexpression in Arabidopsis decreases bicarbonate alkaline tolerance (Zhu et al., 2012a).
*GsWRKY15*	Glyso.05G175600	Glyma.05G207100	group II WRKY	Its overexpression in alfalfa confers bicarbonate alkaline tolerance (Zhu et al., 2017).
*GsWRKY20*	Glyso.08G020100	Glyma.08G021900	group III WRKY	Its overexpression in alfalfa confers salt tolerance (Tang et al., 2014).
*GsbZIP33*	Glyso.03G179600	Glyma.03G219300	subfamily S bZIP	Its overexpression in Arabidopsis decreases salt tolerance (Cai et al., 2011a).
*GsbZIP67*	Glyso.08G248900	Glyma.08G282202	subfamily S bZIP	Its overexpression in alfalfa confers bicarbonate alkaline tolerance (Wu et al., 2018).
*GsNAC20*	Glyso.13G012100	Glyma.13G030900	NAC	Its overexpression in Arabidopsis decreases salt tolerance (Cai et al., 2011b).
*GsNAC019*	Glyso.13G233900	Glyma.13G279900	NAC	Its overexpression in Arabidopsis confers bicarbonate alkaline tolerance (Cao et al., 2017).
*Gshdz4*	Glyso.11G063700	Glyma.11G069400	HD-ZIP	Its overexpression in Arabidopsis confers bicarbonate alkaline tolerance (Cao et al., 2016).
*GsHSFB2b*	Glyso.11G023200	Glyma.11G025700	Class B HSF	It contributes to salt tolerance by promoting flavonoid biosynthesis (Bian et al., 2020).
*GsMYB15*	Glyso.12G163900	Glyma.12G199200	R2R3-MYB	Its overexpression in alfalfa confers salt tolerance (Shen et al., 2018).
*GsZFP1*	Glyso.10G251500	Glyma.10G295200	C2H2 zinc finger	Its overexpression in alfalfa confers salt tolerance (Tang et al., 2013).

Gene ID and ID for *G. max* Homolog were retrieved from the plant genomic resource Phytozome with the *Glycine max* Wm82.a4.v1 and *Glycine soja* v1.1 versions respectively, according to the corresponding information given in the references.

### AP2/ERF transcription factors

The AP2/ERF family TFs are characterized by an AP2 DNA binding domain, consisting of 60 highly conserved amino acids. The AP2/ERF family in plants could be divided into five major subfamilies, AP2, ERF, RAV (Related to Abscisic acid insensitive 3/Viviparous 1), DREB (Dehydration-Responsive Element Binding), and Soloist (Xie et al., 2019). According to a recent study, the soybean genome contains 301 *AP2/ERF* genes, among which 153 were classified into the ERF subfamily (Wang H. et al., 2022a). In *G. soja*, only three ERF subfamily TFs (*GsERF7*, *GsERF71* and, *GsERF6*) have been suggested to participate in salt-alkaline stress response (**Figure 4A**). GsERF7, belonging to the ERF subfamily, was identified as a phosphorylation substrate of GsSnRK1 kinase, and the phosphorylation of GsERF7 on S36 was necessary for its nucleus localization and transactivation activity (Feng et al., 2020). Transformation of *GsERF7* in soybean hairy roots improved the dry weight of composite soybean plants under salt stress. At the same time co-transformation of *GsERF7* and *GsSnRK1* further promoted the tolerance of composite soybean to salt and bicarbonate alkaline stress. *GsERF71* and *GsERF6* also encode ERF subfamily TFs (Yu et al., 2016a; Yu et al., 2017). By overexpressing in Arabidopsis, *GsERF71* and *GsERF6* were demonstrated to specifically confer plant tolerance to bicarbonate (NaHCO_3_ and KHCO_3_) alkaline stress, but not to high pH stress caused by KOH. Both GsERF71 and GsERF6 showed nucleus localization and transactivation activity, and GsERF71 was suggested to bind the GCC box (core sequence AGCCGCC). Interestingly, under bicarbonate stress, *GsERF71* overexpression in Arabidopsis could up-regulate *AHA2* (Arabidopsis H^+^-ATPase 2) expression to facilitate the neutralization of rhizosphere pH and increase the expression of auxin biosynthesis and transporting genes to promote auxin accumulation in roots (Yu et al., 2016a; Yu et al., 2017).

The dehydration-responsive element-binding (DREB) TFs are classified as a subfamily of the AP2/ERF family and have been well-documented to modulate multiple abiotic stress responses. There are 103 *DREB* genes in the soybean genome. Recently, a DREB transcription factor DREB3b was identified to contribute to salt stress tolerance of *G. soja* by combining RNA-sequencing and population genetics analysis (Hou et al., 2022) (**Figure 4A**). Among the 103 soybean *DREB* genes, 24 showed differential expression under salt stress, including 14 up-regulated and 10 down-regulated genes. Out of the 14 up-regulated *DREBs*, 9 displayed natural sequence variation detected by a panel of 424 accessions comprised of 85 wild soybeans, 153 landraces, and 186 cultivars. The natural variation in *DREB3a* and *DREB3b* is statistically related to differences in salt tolerance, and *DREB3b*, undergoes artificial selection during soybean domestication. Soybean plants carrying the *G. soja DREB3b* allele (*DREB3b^39Del^*) are more salt tolerant than those containing the reference genome allele (*DREB3b^Ref^*). Consistently, *DREB3b^39Del^* improved salt tolerance more than *DREB3b^Ref^* in transgenic soybean hairy roots. An InDel marker was developed to identify the *DREB3b^39Del^* allele, and could facilitate the molecular breeding of salt-tolerant soybean varieties in the future. In 2007, Hu et al. also found a Gypsy-like retrotransposon in the 5’ upstream region of *GsDREB1* (GenBank Accession EF051460.1) of individuals among the saline population of wild soybean (Hu et al., 2007). Therefore, these two *DREB* genes are likely targets for molecular breeding to improve soybean salt-alkaline tolerance.

Cai et al. reported that *GsDREB2* deleting the negative regulatory domain (NRD, amino acid residues 140-204) confers salt tolerance in transgenic Arabidopsis, while the full-length *GsDREB2* could not (Cai et al., 2018). The NRD negatively controlled the transactivation ability and DRE element (core sequence A/GCCGAC) binding affinity of GsDREB2, thereby inhibited the function of *GsDREB2* in stress response (**Figure 4A**). Similarly, in Arabidopsis, deletion of the NRD (residues 136 and 165) within AtDREB2A could transform it to a constitutively active form (Sakuma et al., 2006), and the NRD is important for AtDREB2A protein degradation *via* the BPM-CUL3 E3 ligase (Morimoto et al., 2017). In this context, it will be a good choice for soybean breeding through gene manipulation of *DREB2s* by deleting the NRD domain to improve its regulatory effect on salt-alkaline stress tolerance.

### TIFY transcription factors

The TIFY family TFs are characterized by a conserved TIFY motif (TIFF/YXG) and have been demonstrated to widely participate in plant response to various abiotic stresses (Singh and Mukhopadhyay, 2021; Liu W. C. et al., 2022). The TIFY family was divided into *TIFY*, Jasmonate ZIM (JAZ), ZIM-like (ZML), and PEAPOD (PPD) subfamilies. A previous genome-wide analysis identified 34 *TIFY* genes in *G. soja* (Zhu et al., 2013), and a recent study reported 38 *TIFY* genes in *G. max* (Liu X. et al., 2022). In *G. soja*, *GsTIFY10s* (10a, 10b, 10c, 10d, 10e, and 10f) and *GsTIFY11s* (11a and 11b) shared the highest sequence identity and were clustered together in the JAZ subfamily. Notably, all *GsTIFY10/11s* were dramatically up-regulated at the early stage of NaHCO_3_ treatment (Zhu et al., 2013) (**Figure 4B**). Among them, overexpression of *GsTIFY10* (identical to *GsTIFY10a*) and *GsJAZ2* (identical to *GsTIFY10e*) in Arabidopsis improved the tolerance to both salt (NaCl) and bicarbonate (NaHCO_3_) alkaline stress (Zhu et al., 2011; Zhu et al., 2012b). The positive regulation of *GsTIFY10a* and *GsTIFY10e* on bicarbonate alkaline tolerance was further verified by overexpressing in alfalfa (Zhu et al., 2014) and soybean (Zhao et al., 2020), respectively. Recently, a study in *G. max* showed that *GmTIFY10s* expression displayed an apparent response to abiotic stresses, and overexpression of *GmTIFY10e* and *GmTIFY10g* in Arabidopsis and soybean improved salt tolerance (Liu Y. L. et al., 2022). In summary, the *GsTIFY10* genes are key positive regulators of soybean tolerance to salt-alkaline stress. Intriguingly, even though *GsTIFY11b* was highly homologous to *GsTIFY10s*, its overexpression in Arabidopsis resulted in an obvious decrease in salt tolerance (Zhu et al., 2012a). *GsTIFY10s* and *GsTIFY11s* may regulate different signaling pathways under salt-alkaline stress.

Further research is needed to illustrate the regulatory mechanism mediated by *GsTIFY10/11s*. The TIFY proteins generally localize in the nucleus and are considered to be transcriptional repressors even though without a DNA binding domain. Both GsTIFY10a and GsTIFY10e were localized to the nucleus, and neither showed transcriptional activity in yeasts. All *G. soja* TIFY10/11 proteins contain a TIFY domain and a Jas domain. The TIFY domain has been found to mediate homo- and hetero-dimerization of TIFYs and protein interaction with other TFs, such as MYCs. GsTIFY10a could form homodimers or heterodimers with GsTIFY10e. Therefore, screening the interacting TFs of GsTIFY10s is extremely important to understand the *GsTIFY10s*-mediated responsive mechanism under salt-alkaline stress. Besides, more experiments are needed to investigate the background molecular basis to reveal the opposite function between *GsTIFY10s* and *GsTIFY11s* in salt-alkaline stress.

### WRKY transcription factors

The WRKY family TFs are characterized by a conserved DNA-binding domain that harbors the WRKYGQK heptapeptide followed by a C2H2 or C2HC zinc finger. WRKY TFs specifically recognize and bind to the W-Box (core sequence TTGAC) to regulate gene transcription. Several studies have focused on the genome-wide analysis of soybean WRKY genes and their response to abiotic stress (Song et al., 2016; Yu et al., 2016b). The soybean genome contains around 180 genes encoding WRKY transcription factors, which were classified into three groups (Group I-III). Until now, only one *WRKY* gene in *G. soja*, *GsWRKY20* (group III) has been reported to be involved in salt-alkaline stress response (**Figure 4C**). *GsWRKY20* conferred drought tolerance when overexpressing in Arabidopsis *via* an ABA-dependent signaling pathway and by increasing the thickness of the cuticle (Luo et al., 2013a; Tang et al., 2014). Overexpression of *GsWRKY20* and *GsWRKY15* in alfalfa also increased tolerance to salt stress and bicarbonate alkaline stress, respectively (Tang et al., 2014; Zhu et al., 2017). Notably, expression of *GsWRKY20* also resulted in earlier flowering (Luo et al., 2013b), which is an effective strategy for avoiding adverse environmental stress. These studies strongly suggested the multiple regulatory roles of *GsWRKY20* in stress response, which makes *GsWRKY20* a perfect target for soybean molecular breeding to improve the tolerance to diverse abiotic stress simultaneously. In addition, a *G. soja* group III WRKY transcription factor *GsWRKY57* was also suggested to contribute to drought tolerance (Wang et al., 2019a). Considering the high conservation between *GsWRKY20* and *GsWRKY57*, it is possible that *GsWRKY57* also acts as a positive regulator of salt tolerance.

### NAC transcription factors

The plant-specific NAC family is one of the largest TF families, with 226 members in soybean (Hussain et al., 2017). A number of studies have shown that NACs play important roles in the salt-alkaline stress response in plants. Two *NAC* genes in *G. soja* have been suggested to be involved in salt-alkaline stress response. A recent study reported that a NAC family gene *NAC2* functioned downstream of a class B heat shock factor HSFB2b to negatively regulate salt tolerance (Bian et al., 2020). Overexpression of the *NAC2* gene in soybean hairy roots decreased salt tolerance. Under salt stress, *GsHSFB2b* could promote flavonoid biosynthesis by directly inhibiting *GsNAC2*, which repressed the expression of *GsCHS1/CHS4/FLS1/FLS2* (**Figure 4D**). In addition, *GsHSFB2b* can also directly bind to the promoter of *GsC4H* and *GsCHS3* and activate their expression to trigger the flavonoid biosynthesis pathway.

In addition, the Gshdz4-GsNAC019-GsRD29B module was reported to confer tolerance to bicarbonate (NaHCO_3_ and KHCO_3_) alkaline stress but not to high pH stress caused by KOH (Cao et al., 2016; Cao et al., 2017). Gshdz4 binds to the CAATA/TA motif in the *GsNAC019* promoter region and activates the expression of *GsNAC019*; thereby, *GsNAC019* could possibly bind to the CGTA core sequence in the *GsRD29B* promoter (**Figure 4D**). However, further evidence is needed to confirm some details in this module. There is still no genetic data supporting the *GsRD29B* regulation on bicarbonate stress tolerance. Further experiments, for example, EMSA or ChiP-qPCR, are required to verify the binding affinity of Gshdz4 to the *GsNAC019* promoter, and the binding and regulation of GsNAC019 to *GsRD29B*.

It is widely accepted that transcription factors function downstream of protein kinases in the signal transduction network in response to salt-alkaline stress. Phosphorylation is critical for the subcellular localization, DNA binding, and transcriptional activity of transcription factors. Till now, fourteen *G. soja* TF genes have been functionally characterized to participate in salt-alkaline stress response. However, only GsERF7 has been suggested to be directedly targeted and phosphorylated by GsSnRK1. Therefore, identifying protein kinases that interact with and phosphorylate these reported transcription factors in *G. soja* will greatly facilitate to unravel the transcriptional regulation under salt-alkaline stress. Moreover, downstream genes that are directly targeted and transcriptionally regulated by these *G. soja* transcription factors also need to be identified to establish the transcriptionally regulatory network under salt-alkaline stress.

## Conclusions and future prospects

Soybean is one of the most vital beans in the world, providing high-quality vegetable protein, edible oil, and industrial raw materials. Under the background of repeated COVID-19 and the increasing worldwide population, the demand for soybean is increasing day by day. Therefore, it is urgently demanded to improve the soybean production, especially in China. However, its yield is greatly restricted by adverse environmental stresses. Compared with cultivated soybean, *G. soja* retains much higher genetic diversity and displays much higher salt-alkaline stress tolerance. Therefore, *G. soja* is considered as genetic reservoir for breeding new salt-alkaline tolerant soybean cultivars with profitable agronomic traits (Kofsky et al., 2018).

Over the years, researchers have demonstrated that the regulation of ion balance and ROS scavenging is critical for wild soybean to survive on saline-alkaline soil. Genes involved in ion uptake, exclusion, and transport, as well as ROS signaling, and scavenging, have been functionally characterized in *G. soja*. Furthermore, regulatory components of signal transduction pathways, including protein kinases and transcription factors were also revealed. However, these *G. soja* genes were functionally characterized mainly by ectopically expressed in the model plant Arabidopsis or transiently expressed in soybean hairy roots. Considering the potential dysfunction of ectopic expression and functional redundancy of homologues genes, functional characterization of *G. soja* genes in future studies should directly be conducted in transgenic soybean plants.

Although current studies have depicted the roles of some protein kinases, transcription factors and functional genes (such as antioxidant enzymes and ion channels) in salt-alkaline stress, few studies focus on the genetic interplay and protein interaction of these regulators. Therefore, the molecular mechanism and signal pathways of soybean response to salt-alkaline stress are still vague. To facilitate the soybean breeding by multigene pyramiding breeding technology, further studies should be concentrated on characterizing the genetic interplay and protein interaction of these genes to construct the signal transduction pathways of soybean salt-alkaline stress response.

Phytohormones, especially ABA, play key roles in regulating plant tolerance to salt-alkaline stress. Current research has well-illustrated the core components of the hormone signal transduction pathway in plants and has demonstrated the regulation of hormone signaling genes on the tolerance to salt-alkaline stress (Fang et al., 2021; Wang N. et al., 2022b). In *G. soja*, several genes were characterized to modulate salt-alkaline tolerance *via* hormone-dependent signaling pathways. For instance, the expression of ABA-induced genes was up-regulated in *GsWRKY20* and *GsCBRLK* transgenic plants under salt-alkaline treatment (Yang et al., 2010; Tang et al., 2014). *GsTIFY10a* and *GsTIFY10e* mediated the sensitivity to MeJA treatment by suppressing the expression of JA responsive and biosynthesis genes (Zhu et al., 2011; Zhu et al., 2012a). Moreover, *GsERF71* enhanced alkaline stress tolerance by modifying auxin accumulation (Yu et al., 2017). The expression of auxin biosynthesis genes and IAA contents were downregulated by *GsERF71* under alkaline stress. However, how hormone signaling activates salt-alkaline stress response in *G. soja* is still lacking. Therefore, more genetic and biochemical experiments should be conducted to unravel the regulatory mechanism of hormone signaling in *G. soja* response to salt-alkaline stress.

In addition, taking advantage of high-throughput genomic sequencing of hundreds of soybean varieties, more and more attention has been paid to the identification of sequence variations in key genes that regulate salt-alkaline tolerance, for example, *GsCHX1* (Qi et al., 2014), *GsERD15B* (Jin et al., 2021), *GsDREB3b* (Hou et al., 2022) and *GsSALT18* (Guo et al., 2021). Currently, molecular markers of *GsCHX1* have been developed for breeding salt-tolerant soybean cultivars (Lee et al., 2018; Guan et al., 2021). Genes harboring sequence variations between *G. soja* and *G. max* are potential targets for molecular breeding of salt-alkaline tolerant soybean. Therefore, future work should concentrate on identifying salt-alkaline tolerant haplotypes in *G. soja* and re-introduce them into *G. max* to improve the salt-alkaline tolerance.

## Author contributions

Conceptualization, XS and MS. Original draft preparation, XC and BJ. Manuscript review and editing, XS and MS. Funding acquisition, XS and MS. All authors contributed to the article and approved the submitted version.

## Funding

This research was funded by Natural Science Foundation of Heilongjiang Province (grant number JQ2021C002), National Natural Science Foundation of China (grant number 32101672), Special Funds from the Central Finance to Support the Development of Local Universities (To XS, no grant number), and Postdoctoral Science Foundation of Heilongjiang Province (grant number LBH-Q21161).

## Conflict of interest

The authors declare that the research was conducted in the absence of any commercial or financial relationships that could be construed as a potential conflict of interest.

## Publisher’s note

All claims expressed in this article are solely those of the authors and do not necessarily represent those of their affiliated organizations, or those of the publisher, the editors and the reviewers. Any product that may be evaluated in this article, or claim that may be made by its manufacturer, is not guaranteed or endorsed by the publisher.
